# Supplementary material for: Are carbon emissions trading and green financial instruments synergistic? -Comprehensive quantitative research based on content analysis
Source: PLoS One. 2024 Mar 7;19(3):e0298601. doi: 10.1371/journal.pone.0298601 (PMC10919601; doi:10.1371/journal.pone.0298601)
Supplement: S1 Data — (ZIP) [file pone.0298601.s001.zip › Data/地级市Z_F工作报告.zip!/╜¡╬≈/─╧▓2╩╨/└┤╫╘╣1⁄2╓┌║┼í╛┬φ┐╦╩2╛▌═°í┐_2009.docx]

各位代表：

　 现在，我代表市人民政府，向大会作政府工作报告，请予审议，并请各位政协委员和列席会议的同志提出意见。

　 一、2008年工作简要回顾

　 过去的一年，是形势复杂多变、考验异常严峻的一年。在省委、省政府和市委的坚强领导下，全市上下坚持以科学发展观为指导，认真贯彻落实中央和省的大政方针和战略部署，坚定信心，把握大势，善谋实干，攻坚克难，经济社会各项事业取得新成绩,完成了市十三届人大三次会议提出的主要目标任务。

　 （一）成功经受住了多次重大考验。一是年初遭遇了历史罕见雨雪冰冻灾害，全市人民团结一心，组织救灾、抢修人员93.5万人次，努力做到“大灾无大碍、重灾不见灾”，有力保障了全市生产生活秩序的正常运行和社会的安全稳定。二是5·12四川汶川特大地震发生后，我市紧急动员，举全市之力支援灾区抗震救灾及灾后重建工作，社会各界共捐赠款1.55亿元、各类物资价值3300万元；派出医疗、抢险、防疫、电力、公安、建工等救援队伍23批、558人次，赶赴灾区参加抗灾救灾工作，充分体现了南昌人民对灾区同胞的深厚感情和无私奉献。三是5月16日北京奥运火炬接力传递活动在我市隆重举行，在全市人民热情支持和参与下，确保了奥运火炬安全准时顺利传递，未出现任何意外，“南昌传递模式”得到了北京奥组委的高度评价和充分肯定。在北京奥运会和残奥会期间，我市实现了非正常“零上访”和重特大安全生产事故“零发生”。四是积极应对从九月份开始的全球金融危机带来的严重影响和挑战，认真落实国家宏观调控政策和省委、省政府支持南昌做强做大做优的一系列政策，科学调度，精心运作，取得明显成效。

　 （二）主要经济指标继续保持较快增长。全年实现地区生产总值1660.08亿元，增长15%；财政总收入首次突破200亿元，达到230.02亿元，同口径增长14.5%；地方一般预算收入首次突破100亿元，达到102.1亿元，增长17.1%；城镇以上固定资产投资首次突破1000亿元，达到1052.61亿元，增长36%；社会消费品零售总额首次突破500亿元，达到528.89亿元，增长24%；万元GDP综合能耗为0.9158吨标准煤，同比下降4.1%；实际利用外资14.11亿美元，增长14.6%；实际利用内资397.76亿元，增长14.6%；城镇居民人均可支配收入15112元，增长15.6%；农民年人均纯收入5774元，增长14.7%；居民消费价格总水平上升6.1%。

　 （三）重大产业项目顺利推进。招商引资成果进一步扩大。全市新批外商投资项目137个，其中超千万美元（含增资）项目37个；新批内资项目649个，其中超亿元项目46个。32家世界500强企业、33家国内200强企业投资南昌。与IBM、微软在服务外包人才实训方面的合作有了实质性进展。开通了南昌—首尔省内首条国际直航航线，南昌保税物流中心正式获批。工业经济实力进一步增强。全年规模以上工业增加值首次突破500亿元，达543亿元，增长21.3%；实现主营业务收入1770亿元，增长26%；经济效益综合指数达228%，较上年提高17个百分点。“3010”工程和“十百千亿工程”进展顺利，江铃股份小蓝商用车基地、百世德薄膜太阳能电池等6个投资过30亿元的重大项目以及海立压缩机、格特拉克、储科电子等10个投资过10亿元的重大项目开工建设，晶能光电、一元数码、菱光科技、中兴产业园（一期）等重大项目竣工投产。园区经济增长极作用进一步显现，高新区主营业务收入突破500亿元，经开区主营业务收入280亿元，昌东工业园主营业务收入200亿元，小蓝经济开发区主营业务收入180亿元。国有企业改革稳步推进，依法积极稳妥地处理了一批历史遗留问题。农业基础地位进一步巩固。国家级龙头企业发展到9家，省级龙头企业发展到44家。国鸿10万吨肉制品深加工建设加快，启动了国旺、南昌菜园等蔬菜基地建设。蒋巷现代农业示范园、黄马“两江”生态农业走廊、安义现代农业科技园初具雏形。新组建的南昌市农业产业化信用担保公司为29家龙头企业提供融资担保，农村现代流通体系标准化网点已达2500个。服务业水平进一步提升。蓝天碧水购物广场、洪大服装大世界、大润发超市等一批大型商贸中心相继开业，前湖迎宾馆、江西慧谷—红谷创意产业园项目快速推进，青云谱区农副产品市场、青山湖生鲜市场等10家集贸市场得到升级改造。650家“万村千乡”农家店建成，早餐网点覆盖全市。渣打银行南昌分行正式营业，大新银行南昌分行筹备开业，民生银行南昌分行正式获批，南昌市商业银行更名为南昌银行并实现跨区域经营，洪都农村商业银行筹建顺利；与国家开发银行省分行开展第三轮合作，与工行、农行、中行、建行四家省分行签署战略合作框架协议；截止去年年底，全市金融机构存款余额2471.16亿元，比年初增长21.3%；贷款余额2112.02亿元，比年初增长17.9%。编制完成《南昌旅游发展总体规划》，天香园、八大山人梅湖景区、千年古村、宝葫芦农庄、汪山土库等景区景点加快发展，全年实现旅游业务总收入72.49亿元，增长11.9%。成功举办承办中国绿色食品博览会、全国汽车配件交易会、全国制药机械博览会等全国大型品牌会展及中国南昌第七届金秋经贸活动月、“城市发展·南昌论坛”、世界华商领袖年会、2008赣台（南昌）经贸合作研讨会等重大活动，荣获“中国十佳节庆城市”等荣誉称号。

　 （四）县域经济发展强劲。实力不断增强。南昌县财政总收入突破20亿元，并在全省率先跨入全国百强县行列；东湖区财政总收入继南昌县、青山湖区、西湖区之后突破10亿元。农业稳中有升。全年粮食生产面积540.42万亩，增长3%；粮食总产量45亿斤，增长4.2%；生猪饲养量480.25万头，增长3.9%；蔬菜播种面积47.7万亩，产量94.3万吨；水产品起水量32.9万吨，增长5.2%。基础不断夯实。大力实施“造地增粮富民”工程，开发整理土地26.89万亩，改造中低产田6.8万亩；28个渡改桥项目已开工建设9个、其中4个已基本完成；24个国家专项规划内水库除险加固工程已启动16个，12个国家中部四省规划的大型泵站更新改造工程已启动7个。面貌明显改观。乡镇总体规划覆盖率达到100%，行政村建设规划基本完成。对136个自然村进行了公路硬化，全市自然村公路硬化率达54.4%。以南昌县黄马乡获“全国优美乡镇”为标志，在重点镇开展镇容镇貌“五整治、三建设”达标活动，完成629个新农村建设试点。

　 （五）城市品位不断提升。适应城市发展需要，制订了一批专项规划。完成城市综合交通规划、城市轨道交通线网规划的报批工作，编制并上报了城市轨道交通近期建设规划，启动了城市轨道交通1、2号线的可行性研究报告编制工作。编制了城市主要道路过街设施规划、城市重要地段蓝线及重大基础设施黄线规划、城市应急备用水源规划、城市水系综合整治规划、南昌市建设花园城市规划实施方案等。完成八一广场、红谷滩CBD城市设计、南昌水城景观设计整合等八个城市重点地段规划控制要点。完成城北地区、生米大桥以南铁路西客站地区、朝阳洲水厂路以北地区控制性详细规划。完善城市功能，推进了一批基础设施项目。洪都大桥、英雄大桥及连接线工程即将建成，青山湖隧道工程已通车；玉带河主体工程建设与清淤、艾溪湖截污工程、解放东路二期改造、昌东大道一期改造、紫阳大道综合改造、青山湖清淤及东岸整治顺利完成。南昌国际体育中心开工建设。完成了青山北路、井冈山大道、沿江北大道等16处积水点整治改造。南昌铁路枢纽西环线、昌九城际铁路、向莆铁路、铁路西客站、新昌电厂、昌北国际机场扩建、朝阳片区基础设施开发及安置房等工程加快推进；高新南大道(三期)工程、洛阳路隧道工程等项目正式启动；城市轨道交通、天然气进昌项目正在紧张进行前期准备。泉岭垃圾焚烧发电厂项目顺利推进，生活污水处理厂污泥无害化处置项目正式投入运行。文明城市创建成效显著，长效管理机制逐渐完善。按照全国文明城市新的测评体系标准要求，突出抓好创建全国文明城市活动年、六进社区、诚信南昌、为百姓办百件事四项活动，城市整体文明形象得到提升，荣获“全国创建文明城市工作先进城市”称号。注重城管执法重心下移，坚持日常管理与专项整治相结合，推行“城管进社区”，红谷滩新区数字综合城市管理体系一期工程投入试运行。严格土地管理制度，完善土地储备机制，提高节约集约用地水平。注重标本兼治，扎实推进污染减排工作，红谷滩、象湖、小蓝经济开发区污水处理厂正式运行，南昌县、桑海经济技术开发区等一批县城、开发区污水处理厂加快建设；造林绿化“一大四小”工程和国省道环境整治全面启动。城市绿化覆盖率41.04%、绿地率38.6%、人均公园绿地面积8.3平方米；重点企业污染物排放达标率100%；空气质量优良天数344天，优良率93.99%；集中式饮用水源水质达标率100%。

　 （六）人民群众生活持续改善。民生工程顺利推进。全年投入市以上资金17.96亿元，其中省级新增投入4.46亿元,市级新增投入2.21亿元，66件实事得到落实。新增城镇就业10.8万人，其中“4050”人员1.19万人，“零就业家庭”安置率100%，新增转移农村劳动力4.22万人，城镇居民登记失业率3.3%。统筹范围内的离退休人员养老金100%按时足额发放，提高了城市和农村低保人均月补差标准，农村五保集中供养率达80%。城镇职工参加养老保险99.1万人，失业保险53.9万人，基本医疗保险61.2万人，工伤保险61.5万人；城镇居民基本医疗保险61.26万人；参加新型农村合作医疗农民221.64万人，各级财政补贴增加，参合率92.42%；医疗救助57641人次；临时救助3182人次。解决7.1万农村人口饮水安全问题。落实计划生育奖励扶助对象3499人，办理计划生育家庭爱心保险8.4万人。对63.11万名公办义务教育阶段学生免除学杂费和免费提供教科书，对民办义务教育阶段6.04万中小学生补助杂费和教科书费，对贫困学生发放了补助金，完成农村中小学校舍维修改造面积5.8万平方米，消除危房面积5万平方米。为1148名困难群众免费提供了法律援助。廉租房采取租金配租为主、实物配租和租金核减为辅的形式，降低了低保住房困难家庭准入标准（人均使用面积由8平方米提高到10平方米），提高了租金配租标准，全年新增廉租家庭1101户；采取公开摇号方式，有5250户低收入家庭喜得经济适用住房。社会事业全面进步。启动青云谱区广州路学校、西湖区桃花学校、东湖区阳明学校等城区九年一贯制示范学校建设，建立农村中小学校舍维修改造长效机制。加强高层次人才公共服务平台、人力资源市场化服务平台和博士后科研工作站建设。南昌成为全国知识产权工作示范城市，南昌高新区获批中国服务外包示范区、国家知识产权试点园区、国家高新技术产业集群标准化示范基地等三个国字号基地。完成23家乡镇卫生院、226家社区卫生服务机构、380家村卫生所标准化建设。全市人口出生率、自然增长率分别为13.91‰和7.85‰。扎实推进文化大市、文化强市建设，隆重举办了纪念改革开放三十周年小戏小品征集展演、南昌采茶戏周末大戏台、高雅艺术进校园和农民剧团表演等群众文化活动，组建了南昌市英雄艺术团。在韩国成功举办第四届郑律成国际音乐节“南昌日”专场演出。八一起义纪念馆、市博物馆等免费开放；小平小道新陈列馆正式开馆，新四军军部旧址陈列馆改扩建工程正式启动，完成方志敏广场建设。八一起义纪念馆被评为国家一级馆，南昌瓷板画成功申报国家级非物质文化遗产保护项目。城区有线数字电视转换平移基本结束，广播电视综合覆盖率达到98.5%。扎实推行电子政务，“中国南昌”门户网站成功改版，在2008中国政府网站绩效评估中由第81位提升到第22位，“数字南昌”应用系统平台建设进展顺利，成为全国农村信息化综合服务试点城市。竞技体育蓬勃发展，我市运动员在北京奥运会上获1银1铜、残奥会上获1枚金牌，在第六届全国农运会上获8金3银，成功举办“全民健身与奥运同行”等系列群众体育活动和第14届市运会。

　 （七）平安南昌创建扎实推进。坚持开展普法教育和依法治市工作，加强社会治安综合治理，深入开展打黑除恶、命案侦破、打击“两抢一盗”、非法河道采砂等专项行动，严厉打击各类违法犯罪行为；全面加强“两所一庭”和“三基”工程建设，落实屯警街面措施，群众安全感指数达到95.26%，被授予全国“科技强警示范建设城市”。切实加强安全生产、消防安全、食品药品安全、森林防火安全，加大交通安全专项整治力度，改善道路交通条件，推进道路畅通工程。在市长热线基础上，开通市长手机（信箱），拓宽民生通道；高度重视信访和人民调解、司法调解、行政调解工作，积极开展市直机关干部下访督导，化解了一批矛盾纠纷；加强突发事件应急处置，成功举办“应急2008”自然灾害综合应急演练和2008中国南方突发重大动物疫情应急演练，不断提升各级各部门的应急处置能力。

　 （八）政府自身建设不断加强。加大立法工作和执法监督力度，提请市人大常委会审议地方性法规4件，制定和修改政府规章6件，出台规范性文件6件；推行行政执法责任制，开展行政执法评议考核，审结行政复议案件153件，维护了公民、法人和其他组织的合法权益。广泛听取社会各方面的意见，认真办理人大代表议案、建议和政协委员提案，坚持政务公开和科学民主决策。以“抓效能、促项目、兴发展”为主线，在全市范围内开展“深化机关效能建设和优化投资环境‘双月整治’”活动和民主评议政风行风活动。整合部门行政审批职能，将行政审批职能归并集中到专设的行政审批服务处，行政审批服务处成建制集中进驻市行政服务中心，实现审批服务事项进驻到位、向窗口授权到位和窗口负责人配备到位。整合市建设工程交易中心、产权交易中心、政府采购中心职能，组建江西省南昌公共资源交易中心；加大纠风治乱和效能监察力度，开展专项治理，切实解决损害群众利益的突出问题。率先在全省对行政审批流程再造，大大压缩行政审批环节和时限。认真规范和严格落实重大项目跟踪推进、协调调度、督查问效和信息共享平台制度，不断优化投资和发展环境。

　 与此同时，大力开展国防教育，加强人防建设，推进优抚安置和双拥工作；外事侨务工作成绩显著，新缔结国外友好城市两对；民族宗教、对台事务工作继续加强；统计、审计、物资、参事、档案、地方志、保密、老龄、妇女儿童、残疾人、消费者权益保护、防震减灾、科学普及、质量技术监督等工作，都取得了新的成绩。

　 各位代表，面对国内外复杂多变的形势，南昌经济社会发展能取得这样的成绩，确实来之不易。这是全市人民在市委的正确领导下，认真贯彻党中央、国务院和省委、省政府大政方针，众志成城、共克时艰的结果；是市人大依法监督和市政协民主监督的结果；是各民主党派、工商联、无党派人士、各人民团体和驻昌单位、人民解放军、武警官兵以及离退休老同志共同努力、大力支持的结果。在此，我谨代表市人民政府，向全市人民，向所有关心、支持南昌发展的同志们、朋友们，表示衷心的感谢并致以崇高的敬意！

　 在总结2008年工作的同时，我们也清醒地认识到，与沿海发达城市和中部省会城市相比，我市的经济总量还不大，城市综合竞争力还不强。主要表现在：具有较高科技含量和市场占有率的企业和产业集群还不多，城乡规划建设管理水平还有待进一步提升，扩大就业、促进群众增收的压力增大，节能减排和环境保护任务十分艰巨，影响社会稳定的因素还不少，政府效能仍需进一步提高。对此，我们将进一步采取措施，认真加以解决。

　 二、2009年的工作

　 今年是新中国成立60周年，也是应对国际金融危机冲击、促进南昌快速崛起的关键之年。当前，由美国次贷危机引发的国际金融危机尚未见底，还在继续蔓延，全球经济衰退的风险越来越大，对我国、我省、我市经济的直接和间接影响已经显现并逐步加重。严峻的外部经济环境与长期制约我国经济发展的体制性、结构性矛盾并存，经济增速下滑的压力加大，今年经济运行将面临更加困难的局面。

　 面对严峻的挑战，我们要冷静审视、把握大势，既要看到不利因素，更要看到有利条件。一是翻开经济发展史，但凡每一次大的经济危机，也孕育着巨大的机遇，每一次大的经济波动期，也是资金、人才、技术的加快流动期。只要善于捕捉和把握机遇，就有可能在经济波动中加快赶超的步伐。二是改革开放以来，尤其是进入21世纪以来，南昌经济发展的聚集力、辐射力明显增强，加上外经外贸依存度相对较低、区位优势明显等独特优势，为抵御风险和承接产业梯度转移提供了有利条件。三是党中央、国务院准确把握形势，迅速出台一系列扩大内需、促进经济增长的政策，省委、省政府出台了支持南昌做强做大做优的一系列举措，为我市保持经济平稳较快增长提供了重要的宏观环境。四是省委、省政府提出建设鄱阳湖生态经济区的战略构想，是一项加快区域经济发展的宏大工程，作为鄱阳湖生态经济区核心城市的南昌，将迎来经济社会发展的巨大机遇。五是2011年第七届全国城市运动会在南昌举办，对促进南昌经济发展、完善城乡功能、提升现代文明搭建了新的历史平台。只要我们科学把握当前形势，充分发挥自身优势，进一步调动广大干部群众的积极性和创造性，凝聚全社会的智慧和力量，坚定信心，善谋实干，我们就能从国际国内条件相互转化中抢抓发展机遇，从国际国内资源优势互补中创造发展条件，就能变压力为动力，变危机为生机，变经济波动期为发展机遇期。

　 2009年政府工作总体要求是：全面贯彻落实党的十七大、十七届三中全会、中央经济工作会议、省委十二届八次全会和市委九届六次全会精神，深入学习实践科学发展观，按照胡锦涛总书记视察江西时提出的“大力弘扬井冈山精神，全力以赴保增长、千方百计保民生、加大力度保稳定”的要求，朝着“六个方面走在全省前列”目标，全力做好 “两篇文章”，坚定不移地实施大开放主战略和新型工业化核心战略，奋力推进经济大发展、城乡大变样、文明大提升三大工程，确保经济平稳较快发展，为2011年第七届全国城市运动会的顺利召开打下坚实基础。按照上述总体要求，今年全市经济社会发展的主要预期目标是：

　 地区生产总值增长13%；

　 规模以上工业增加值增长18%；

　 财政总收入增长15%；

　 地方一般预算收入同口径增长15%；

　 全社会固定资产投资增长32%；

　 社会消费品零售总额增长16%；

　 实际利用外资增长10%；

　 实际利用内资增长10%；

　 城市居民年人均可支配收入增长9%；

　 农民年人均纯收入增长9%；

　 单位生产总值能源消耗比上年下降4%；

　 人口自然增长率控制在7.9‰以内；

　 城镇居民登记失业率控制在4.5%以内；

　 居民消费价格总水平涨幅控制在4个百分点左右。

　 完成上述预期目标，压力和挑战是巨大的，但也是积极可行的，符合加快发展、科学发展的要求，与中央关于保增长的要求相一致，也与全市“十一五”规划相衔接，只要我们统一思想，坚定信心，扎实工作，经过艰苦努力是可以实现的。为此，我们将突出抓好以下工作：

　 （一）以重大项目推动为突破口，不断推进“一个基地、三个中心”建设

　 强化工业主导地位，全力推进先进制造业重要基地建设。进一步明确产业定位。坚持科技强市，用高新技术改造传统产业，大力发展高新技术产业。以高新、经开两个国家级开发区和小蓝经济开发区为重点，支持各开发区打造特色、差异发展、扬优成势。围绕一园（承接台资企业转移基地和台商创业园）、两基地（光伏产业基地和航空产业基地）、三产业（半导体照明、汽车及零部件、服务外包产业）、四重点（机电制造、冶金和新材料、医药食品、电子及电子信息产业），继续抓好“3010”工程和“十百千亿工程”。进一步实施重大项目推动战略。重点打造光伏、LED、汽车及汽配3个主营业务收入可望超千亿元的产业，以及电子信息、新型材料、机电制造、医药食品4个主营业务收入可望超500亿元的产业，壮大一批主营业务收入超50亿元的龙头企业。完成江铜—耶兹铜箔公司高档电解铜箔（二期）改造、江钨集团公司金属镍钴、江西格特拉克变速器、海立空调压缩机、人民电器等21个项目，续建江铜集团铜板带、晶能光电蓝/绿光芯片、泰豪科技公司中低压发电机制造基地等32个项目，开工建设洪都航空城、江西赣江海螺水泥等11个项目，加快推进长力钢铁公司转炉厂技术改造二期、晶能光电公司照明产业化等9个项目的前期工作。进一步加大政策扶持力度。整合财政专项资金，加大财政投入，设立扶持企业发展专项资金，用于扶持重点企业和重大项目。按照“重点项目保障用地、优质项目优先供地、所有项目节约集约用地”思想，拓展发展用地空间。着力实施高级管理、高级技术、服务外包“三个一万”人才工程。进一步推进市级工程中心、重点实验室建设，加快瑶湖生态科技城建设。增加中小企业发展专项资金和全民创业专项资金规模，构筑多层次、多渠道中小企业融资体系，提升非公经济发展水平。进一步加强运行调度。密切关注国际、国内市场动态，对全市工业经济逐月进行监控，重点加强对全市工业经济影响较大的重点企业产、销、存的跟踪分析，科学分析工业经济运行现状及发展态势。全面实施好增值税转型改革，减轻企业负担，保持企业生产成本竞争优势，支持企业扩大市场占有率。

　 大力发展现代服务业，加快推进商贸、物流、金融中心建设。优化商贸业布局。稳步提升老城区市级商业中心水平，加快推进红谷滩中央商务区商贸服务设施建设。继续推进城东、城南、城西、城北区级商业中心的规划建设。继续巩固和完善社区商业网点。加快物流业发展。积极推进南昌保税物流中心和昌北航空、向塘铁路等物流中心建设，加快推动华中国际物流城建设。建成南昌市大中专院校放心粮油连锁配送中心项目，续建江西慧谷—红谷创意产业园、南昌市服务外包产业基地等项目。提高金融业集聚能力。支持在昌金融机构扩大业务、防范风险；力争再引进1—2家商业银行；加快大新银行南昌分行、民生银行南昌分行开业和南昌银行上市步伐，加快组建洪都农村商业银行，推进红谷滩金融大街建设。全面启动红谷滩总部基地一期工程。提升旅游会展业品牌。突出红色摇篮、绿色家园主题，落实旅游发展规划，挖掘南昌文化底蕴，推进滕王阁二期改造，加快天香园、八大山人梅湖景区等重点景区开发建设。加强国际性、区域性旅游合作与交流，强化城市旅游形象的宣传促销，开发具有地方特色的旅游纪念品。大力发展会展经济，积极申办世界低碳与生态经济大会暨技术博览会、中国绿色食品博览会、中国服务外包博览会等国际国内大型会展。

　 （二）以开展统筹城乡一体化试点工作为契机，不断推动农村发展和农民增收

　 壮大县域经济实力。抓紧制定统筹城乡经济社会发展一体化试点工作方案，坚持“工业反哺农业，城市反哺农村”方针，加快开展试点工作。进一步发挥比较优势，扶持县区工业园加快发展，打造特色鲜明的经济板块，培育壮大经济强县。落实各项强农惠农政策，继续抓好优质粮食产业工程，稳定面积保总量，提高单产扩增量。加快发展畜牧业，强力推进特种水产养殖业和以蔬菜、水果、苗木和花卉为主的种植业发展。继续大力实施农业产业化“双十双百双千” 工程，精心运作农业产业化信用担保公司，培育一批示范型专业合作社，促使更多的龙头企业与农户建立紧密型利益联结机制。切实完善农村现代流通体系，提高农产品竞争力和市场占有率。加快农业科技成果推广，积极推进农业机械化，千方百计促进农民增收。

加强农村基础建设。大力推进以优质粮食工程为重点的农田水利基本建设，重点实施水库除险、泵站改造、渠系整治等工程。一年内完成专项规划内病险水库除险“摘帽”任务，两年内完成列入国家中部四省规划的大型泵站更新改造。加大以改造中低产田和建设标准农田为主要内容的农业综合开发力度，整合涉农项目，推进规模农业综合开发，提高农业综合生产能力。强力推进农村环保小康行动，续建南矶山湿地自然保护区湿地保护建设项目，白水湖水系防洪排涝综合整治工程。全面实施水土保持生态修复建设，完成治理水土流失面积11万亩。积极实施造地增粮富民工程，确保土地利用占补平衡。

　 加大社会主义新农村建设力度。认真落实城镇建设规划，继续推进国省道沿线及乡镇集镇环境卫生和村社环卫保洁、村庄绿化示范区建设。按照省、市、县三级共建的方式，大力推进650个左右社会主义新农村建设试点村的“六改四普及”、“三绿一处理”和“三化（划）三改一习惯”的创建工作，推进中小学危房改造、卫生院改扩建等基本建设。大力推进农村信息化建设，提高农村信息服务水平。建立和完善农村防灾减灾服务体系，建设农村气象灾害预警信息发布系统，提升农村社会事业发展水平和公共服务能力。

继续深化农村改革。进一步加大财政体制调整力度，推进乡财乡用县监管改革，完善县乡财政管理体制。贯彻落实基本农田保护“五不准”和耕地保护责任制，切实保护基本农田。稳定和完善农村基本经营制度，严格按照“依法、自愿、有偿”的原则，积极稳妥地探索有利于土地流转的形式和途径，建立完善的流转激励机制。建立健全稳定增长的农业投入机制，建立和规范农村金融机构，大力发展小额信贷和微型金融服务。完善主要农产品最低收购价制度，切实保障农民利益。继续推进集体林权制度配套改革、小型农业水利产权改革，适时推进国有农场管理体制改革，不断激发农村发展活力。

　 （三）以筹办“七城会”为抓手，不断提升城市综合承载能力

　 强化规划“龙头”作用。坚持规划高起点，继续编制完成《南昌市城市发展战略规划研究》，完成市县乡三级土地利用总体规划修编，城市轨道交通1、2、3号线可行性研究报告及沿线用地控制性详细规划、西客站综合交通规划，启动编制开发区控制性详细规划，研究扬子洲地区保护控制规划，组织编制南昌市人民防空建设规划、南昌市历史文化名城保护规划、象湖西岸规划等。

加强基础设施建设。坚持建设高标准，继续推进“一江两岸”工程，切实加快以交通、能源、水利等为重点的工程项目建设。完成洪都大桥、英雄大桥、高新南大道三期、昌东大道二期改造、洛阳路隧道等续建工程；继续推进向莆铁路、昌九城际、南昌西外环、昌北机场扩建等项目建设；启动洛阳路、青山湖南大道、解放东路延伸段、艾溪湖大桥等项目建设，争取南外环、南昌至奉新高速公路建设开工，做好昌吉赣城际铁路各项前期准备工作。全面展开朝阳片区三纵三横主路网和罗家组团二纵三横主路网建设。全力推进城市轨道交通项目建设。加快新昌电厂建设步伐，争取年内第一台机组建成投产。加快天然气管网建设步伐，争取年内供气。加快污水处理厂收集管网系统建设步伐，提升青山湖污水处理厂一期功能，适时启动二期工程。加快玉带河附属工程建设，完成朝阳水系整治，推进乌沙河改造项目开工。抓紧南昌国际体育中心、射击中心、水上运动中心和前湖迎宾馆等相关接待设施建设，续建朝阳公园工程。

提升城市管理水平。深入开展以“和谐交通、文明出行”为主题的道路畅通工程，着力完善老城区快速交通干道系统，改善次干道和支路系统，科学组织交通微循环，提高现有道路通行能力。深入开展“绿色生态南昌建设”专项行动，限期完成重点企业的环保设施改造工程。加大植树造林和道路绿化力度，改善路容路貌，提高绿化率。大力开展爱国卫生运动，巩固和发展“国家卫生城市”成果。坚持“既要金山银山，更要绿水青山”，以南昌成为全国“节能与新能源汽车示范推广试点城市”为契机，严格节能降耗工作责任制，打好污染减排攻坚战，开展清洁生产，发展循环经济，培育新能源汽车产业。积极开展“城市精细化管理年”活动，加大城市规划的执行力度，建立健全条块结合、以块为主的长效管理机制。全面推动南昌数字城管建设，组建市级城市管理长效机构。

　 （四）以改革开放为动力，不断增强开放型经济和体制机制创新优势

大力发展开放型经济。坚持推进产业招商与优化产业结构并举，推动内外资、先进技术向支柱产业和重点企业集中，向先进制造业、现代服务业聚集，向现代农业领域延伸。同时，以现有龙头企业为支撑，突出开展配套招商，形成产业集聚效应。积极参加省政府组织的招商推介活动，承办好第七届赣台会。精心组织全市重大招商引资活动，办好第八届金秋经贸活动月。积极转变外贸增长方式，优化出口产品结构，紧紧抓住南昌被国家批准为中国服务外包示范城市的有利时机，加大服务外包培训力度，主动承接国际服务外包业务，发展软件和信息服务外包产业，尽快把南昌建成全国重要的服务外包示范基地。稳妥推进我市企业跨国经营，支持江铃在阿尔及利亚建设中国江铃经济贸易合作区。

着力推进机制体制创新。继续推进国有企业改革攻坚，通过股权置换、整体转让、改制上市和引进战略投资者，实现投资主体多元化。稳妥推进企业上市、市昌工控股资产重组、省属下放企业改制。继续实施已列入国家政策性破产的11家企业破产工作。进一步完善商业用地净地出让制度和工业用地招拍挂制度，积极探索建立污染物排放总量控制制度、排污权交易制度。加强政银企合作，做强做大市城投公司、市市政控股集团、市轨道交通公司三大投融资平台。

　 （五）以民生工程为重点，加快发展社会事业

　 继续坚持新增财力向基层倾斜、向农村倾斜、向困难群众倾斜、向社会事业倾斜，推动以62项民生工程实事为重点的各项社会事业发展。

努力扩大就业和促进创业。继续把就业再就业作为一号工程来抓，高度重视返乡农民工就业问题，通过落实国家支持就业政策和经济发展、政府补贴、培训服务等形式，提高就业水平。重点办好5件实事：进一步扩大就业，全市城镇新增就业7.04万人；加快农村富余劳动力转移，新增转移农村劳动力3万人；加强就业培训，免费培训7万农村劳动力；扩大小额担保贷款规模，完成小额担保贷款2.1亿元；帮助残疾人就业，残疾人就业率达到66%。

　 继续完善社会保障体系。重点办好5件实事：提高企业退休人员养老金水平；扩大养老保险覆盖范围，全市城镇职工参保人数增加5万人；推进被征地农民养老保险试点，依据国家政策适时出台农民工养老保险办法，参保人数达到1.54万人；提高失业保险统筹水平，参保人数增加2万人；扩大城镇职工工伤保险覆盖范围，参保人数增加0.3万人。

　 不断提高社会救助水平。重点办好11件实事：提高城乡低保对象补助标准；提高农村五保户供养标准；提高上世纪六十年代精简退职老职工救济金水平；妥善安置政策性、市属破产企业和困难企业职工生活；提高城镇“三无”特困群众供养标准；建立农村困难群众住房救助制度；建设市级流浪未成年人救助保护中心、南昌市儿童福利院；继续实施计划生育奖励制度；适当提高义务兵优待金标准和退役士兵自谋职业一次性补助水平；安排基层司法法律援助专项资金，加大城区司法所建设力度等。

　 大力加强医疗保障和食品药品安全。以建设“健康南昌”为目标，合理布局卫生资源，增设新区医疗卫生服务网点；加强疾病预防控制和市血站能力建设；加快农村卫生和社区卫生发展，逐步建立“分级保障、低水平、全覆盖、医疗救助、特殊人群救助”的全民医疗保障体系。重点办好13件实事：城镇职工基本医疗保险参保职工达到62万人，全市城镇居民和大学生全部纳入城镇居民基本医疗保险范围；推进新型农村合作医疗制度建设，参合率接近95%；将城乡低保户、城乡低保边缘户、农村五保户和新增四类人员纳入城乡医疗救助范围；儿童国家免疫规划疫苗免费接种率达到100%；推进城镇居民公共卫生服务制度建设；关心残疾人；开展“取消医院药品加成”试点；建立村级医生公共卫生服务补助制度；加强食品药品安全检测和监管工作；县、乡、村、社区卫生服务机构服务能力逐步达到国家建设标准等。

　 优先发展教育事业。新增教育经费重点支持农村学校建设，加大城区九年一贯制义务教育示范学校建设力度，新建改造一批市、县区特殊教育学校，新建两所优质高中，完善“一核五片”职教园区建设，建设一批示范专业和示范院校。重点办好4件实事：增加城乡义务教育发展经费，支持城市义务教育阶段“大班改小班”建设，支持农村中小学配套设施和农村中学实验室建设，向城乡义务教育阶段的学生免费提供地方课程通用教材；扩大家庭经济困难学生资助范围；资助城乡困难家庭子女免费入读技工学校；加大城乡义务教育教师津补贴支持力度。

积极发展文化、体育等事业。重点办好7件实事：继续推进农村文化“三项活动”；提高农村宽带网络覆盖率，达到100%；加大对文物和非物质文化遗产的保护；加强农村文化设施建设；向公众免费开放博物馆和纪念馆；加强基层体育健身场所建设；建立居委会工作人员补贴制度和敬老院工作人员补贴制度。

　 加大保障性住房建设力度。坚持对低收入住房困难群众实行住房保障制度，加大政策支持力度，加快保障性住房建设。重点办好3件实事：扩大廉租住房保障范围，增加实物配租；继续开展经济适用住房建设，新增33万平方米；积极推进低洼地危房改造。同时，贯彻落实国家稳定房地产市场政策，加强房地产市场监测分析，促进房地产市场健康发展。加强城区物业管理，提升全市物业管理行业水平，完善功能配套。

　 大力推进扶贫开发。重点办好3件实事：继续抓好整村推进扶贫开发；积极开展农业产业化扶贫；启动小水库移民解困工作。

　 改善人民生产生活条件。重点办好11件实事：加大农村居民饮水安全，解决27.5万农村人口饮水安全问题；新增农村沼气用户5804户；实施造林绿化“一大四小”工程；推进650个新农村建设试点村的农民家庭生活垃圾处理工作；扩大农业政策性保险试点和农机具购置补贴范围；提高公益林补偿标准；加大农村电网改造力度；加强农村交通基础设施建设；实施便民出行、环境美化等工程；改造10—12家集贸市场；建设和改造“农家店”600家。

　 （六）以构建和谐文明南昌为目标，不断营造文明向上、安全有序的社会环境

　 不断提升现代文明。大力弘扬“大气开放、诚信图强”的城市精神，继续推进“全国文明城市”创建，积极培养公平正义、诚信友爱、健康向上的公民道德，全面提升市民素质。着眼建设文化大市、文化强市，加快文化事业发展。着力推进实施乡镇综合文化站工程、文化信息资源共享工程、农村电影数字化放映工程和一批示范社区文化中心建设。启动无线城市建设。全面推进县（区）有线数字电视转换。高质量完成中国新四军陈列馆工程，启动南昌市群众艺术馆和八大山人纪念馆真迹陈列馆建设。大力繁荣文艺创作，打造文艺精品。办好第十八届金鸡百花电影节和第三届南昌国际军乐节。推动文化体制改革，扶持南昌英雄艺术团等文艺团体发展。加快报业大厦建设；深入开展“扫黄打非”工作和“反盗版天天行动”，举办“和谐南昌”系列文艺展演活动，营造健康向上、文明和谐的文化环境。继续开展普法教育。全面实施“五五”普法和依法治市规划，积极开展“法治南昌”创建活动，推进基层民主和法制建设，加强法律援助工作，不断提高公民的法律意识，依法保护公民的合法权益。强化社会治安综合治理。推进新一轮平安南昌建设，力争用两年时间建立初步覆盖城乡的视频监控体系。继续加大对基层司法所建设投入，推进公安信息化建设、执法规范化建设及和谐警民关系建设，依法严厉打击各种刑事犯罪，切实增强人民群众的安全感。有效化解各种社会矛盾。高度重视信访工作，完善信访制度，落实领导信访责任制和“一岗双责”制，在源头治理、化解矛盾上下功夫，最大限度地把问题解决在基层、解决在萌芽状态。毫不松懈抓好安全生产。更加严格地落实好安全生产领导责任制和事故责任追究制，加强对重点行业、重点领域的安全生产综合监管和执法力度，加大排查和治理事故隐患力度，坚决防止重特大安全事故的发生。着力提升应急处置能力。进一步完善和落实应急预案，建立健全各级应急管理领导机构、办事机构和工作机构，落实应急管理责任制，抓好应急预案演练，加强各级各类应急队伍建设，妥善应对各类突发公共事件，保障人民群众生命财产安全，切实维护安定团结、和谐稳定的局面。

　 复杂多变的形势和挑战，是对政府行政能力的重大考验。越是在宏观经济环境困难的情况下，我们越要增强宗旨意识、责任意识、忧患意识、机遇意识、服务意识和节俭意识。要以开展“深入学习实践科学发展观”活动为契机，自我加压，负重奋进，更好地肩负起执政为民、富民兴昌的历史重任。一是坚持依法行政。进一步加强政府立法工作，规范行政执法行为，推进综合执法改革，强化行政执法监督。加强行政效能监察，严格执行行政问责制，拓宽效能投诉渠道，做到有权必有责、用权受监督、侵权要赔偿、违法必追究。自觉接受人大及其常委会的依法监督和政协的民主监督，自觉接受群众监督和舆论监督。认真办理人大代表议案、建议和政协委员提案，重视和发挥各民主党派、工商联、无党派人士和群众团体的作用。二是提高行政效能。按照省开展“机关效能年”、市开展“项目操作年”活动的要求，进一步削减行政审批事项，规范办事程序，减少办事环节，落实行政审批“两集中三到位”。大力推进电子政务建设，着力推进市县（区）、开发区行政服务中心电子监察系统建设，建立市县（区）审批办证联网的统一平台，逐步推行网上审批，完善重大项目绿色通道服务制度，创优“四最”品牌。始终坚持群众路线，深入基层，真诚倾听群众呼声、真情关心群众疾苦，急群众所急、想群众所想；进一步倡导求真务实之风，坚持问政于民、问需于民、问计于民，说实话、干实事、求实效，真正把精力放在谋划发展上，把功夫下在为民惠民上，做到权为民所用、情为民所系、利为民所谋。三是保持清正廉洁。坚持执政为民，从严治政，继续加强公务员队伍建设。从教育、制度、监督、惩处、改革等方面入手，认真落实惩治和预防腐败体系建设各项工作，形成拒腐防变的长效机制。严格执行领导干部廉洁从政各项规定，加强作风建设，进一步落实党风廉政建设责任制，筑牢思想道德防线。完善公共资源交易中心监督管理，进一步强化公共资源交易工作；加大对财政性投资的监督管理力度，深化商业贿赂治理，坚决纠正损害群众利益的不正之风，依法查处违法违纪案件。倡导勤俭节约，杜绝浪费行为，努力降低行政成本。

　 各位代表，2009年是在新的起点上开启南昌改革开放新征程的一年。让我们更加紧密地团结在以胡锦涛同志为总书记的党中央周围，在省委、省政府和市委的正确领导下，全面落实科学发展观，团结一心、锐意进取，全力推动新一轮经济大发展、城乡大变样、文明大提升，以优异成绩向共和国60华诞献礼！
